# The Potential Roles of Pharmacists in the Clinical Implementation of Pharmacogenomics

**DOI:** 10.3390/pharmacy11060180

**Published:** 2023-11-19

**Authors:** Abdullah Al Maruf, Md. Abdul Aziz

**Affiliations:** 1 College of Pharmacy, Rady Faculty of Health Sciences, University of Manitoba, Winnipeg, MB R3E OT5, Canada; azizma@myumanitoba.ca; 2 Bangladesh Pharmacogenomics Research Network (BdPGRN), Dhaka 1219, Bangladesh; 3 The Mathison Centre for Mental Health Research & Education, Hotchkiss Brain Institute, University of Calgary, Calgary, AB T2N 4Z6, Canada; 4 Department of Psychiatry, Cumming School of Medicine, University of Calgary, Calgary, AB T2N 4N1, Canada

The field of pharmacogenomics is at the forefront of a healthcare revolution, promising to usher in a new era of precision medicine. The intricate interplay between genes and medications lies at the heart of pharmacogenomics, offering the potential to enhance the safety and effectiveness of pharmacological treatments [1,2,3]. Despite a number of challenges, pharmacogenomic testing is already being used in over 70 healthcare systems worldwide, and commercial providers presently offer a variety of options, including direct-to-consumer tests [4,5,6,7,8,9,10]. Pharmacists are medication experts and the most accessible healthcare professionals to the general public. Their understanding of pharmacokinetics and pharmacodynamics makes them uniquely prepared to interpret the results of pharmacogenomic tests [11,12,13,14,15]. The widespread accessibility of pharmacists also grants a strategic position from which to drive the practical adoption of pharmacogenomics in clinical practice. Nearly all pharmacy programs in North America have added pharmacogenomics content to their curricula after the Centre for the Advancement of Pharmacy Teaching recommended that it be included in pharmacy education in 2016 [16,17,18]. “Pharmacogenomics” content is included in the accreditation standards of both the Canadian Council for Accreditation of Pharmacy Programs and the Accreditation Council for Pharmacy Education, USA [19,20]. Despite having a positive attitude toward pharmacogenomic testing, pharmacy students showed low confidence in their preparation to use this knowledge in their practice, partly due to varying content and learning methods [21,22,23,24]. In a position statement from 2022, the ASHP (American Society of Health-System Pharmacists) emphasized pharmacists’ responsibilities in the clinical application of pharmacogenomics [25]. The feasibility of pharmacogenomic testing in pharmaceutical practice is well-documented in various implementation trials [26,27,28,29,30,31]. Yet, few pharmacists feel they have the appropriate knowledge base to provide this service confidently to their patients or discuss testing results with other healthcare providers [32,33,34].

Recent advancements in the pharmacogenomic sector highlight the growing importance of pharmacists in related clinical practice and research [35]. This Special Issue includes five original studies and a review of the growing involvement of pharmacists in the clinical use of pharmacogenomics. Haga SB [36] summarized the various roles pharmacists play in the clinical application of pharmacogenomics, particularly in the USA. The author also highlighted the leadership of pharmacists in this field and talked about potential obstacles and future directions. The author also correctly noted that the effective delivery and appropriate application of pharmacogenomic testing in various clinical contexts depend on pharmacists and prescribers working together. With the advancement of pharmacogenomic research and technologies, including the use of artificial intelligence, there is no doubt that pharmacists’ educational needs in pharmacogenomics will continue to evolve.

The feasibility and functioning of a cooperative circuit, combining hospital and community pharmacists collaborating with geneticists and clinicians in order to adopt clopidogrel pharmacogenetic testing, were assessed in a prospective trial from Spain by Mir and colleagues [37]. In addition to improving the circuit’s overall utility and patient and healthcare provider satisfaction, this cooperative effort increased some of its operational elements. It also set a positive precedent for future endeavours.

Another prospective single-centre interventional study from the Netherlands by Kerskes and colleagues [38] involving polypharmacy patients with chronic kidney disease evaluated the utility of pharmacogenomic testing in regular medication evaluations. Hospital pharmacists and nephrologists collaboratively conducted pharmacogenomic profiling and automated medication surveillance to identify gene–drug interactions and determine the clinical significance and the need for pharmacotherapeutic interventions. The study demonstrated the potential benefits of optimizing pharmacotherapy for chronic kidney disease patients, suggesting that pharmacogenomic testing could enhance routine medication assessments.

A cross-sectional web-based survey by Hayashi and Bousman [39] conducted in Alberta (a province in Canada) hospitals assessed healthcare providers’ (pharmacists, nurse practitioners, and physicians) knowledge, experience, and perceptions of pharmacogenomics. The study found that a significant proportion of the respondents lacked knowledge, training, and exposure to pharmacogenomics. Despite limited knowledge, participants expressed positive attitudes toward the clinical usefulness of pharmacogenomics, particularly in oncology, cardiology, and psychiatry. Barriers to implementation included knowledge gaps, cost considerations, and a lack of guidelines and evidence. The study results provide a guideline for future initiatives in Alberta and other regions in Canada to promote the awareness of the accessibility and reliability of evidence for using pharmacogenomic testing in optimizing patient care.

Stäuble and colleagues [40] from Switzerland developed a structured pharmacogenomic testing and counselling service to be offered by pharmacists based on observational study data. This was in response to the challenging process of integrating pharmacogenomics into clinical practice. The essential components of the pharmacogenomic testing service included patient referrals, pharmacogenomic testing, medication reviews, pre- and post-test counselling, and follow-up. These steps highlight the importance of interprofessional collaboration among healthcare providers and pharmacists’ role in an interprofessional healthcare environment.

A cross-sectional study from Croatia by Bukic and colleagues [41] compared the attitudes of biomedical students in various healthcare branches to the utilization of pharmacogenomics in clinical practice. The students acknowledged the advantages of applying pharmacogenomics in clinical practice and showed a keen interest in its clinical application. The majority of students said that their future practices require the ability to recognize patients who are at risk and could benefit from pharmacogenomic testing. The study emphasized the importance of educational institutes in incorporating more pharmacogenomics education into their curricula and stressed the need for continuing education in pharmacogenomics for healthcare professionals. The study also emphasized that ethical concerns related to the implementation of pharmacogenomics should be addressed and regulated to ensure its appropriate and ethical use in practice.

These international studies have demonstrated the potential roles of pharmacists in the clinical implementation of pharmacogenomics worldwide. We expect the following from pharmacists (adapted from the ASHP position statement [25]) (Figure 1):

(1) Pharmacists should lead in the clinical implementation of pharmacogenomics as drug–gene interaction experts.
(2) Pharmacists should use pharmacogenomic testing for appropriate patient cases and use the test results to optimize medication therapy.
(3) Pharmacists should be the point of contact for patients and healthcare professionals in terms of interpreting pharmacogenomic test results and providing educational resources.
(4) Pharmacists should be able to confidently provide one-to-one consultations on pharmacogenomic test results to their patients.
(5) Pharmacists must collaborate with healthcare providers from multiple disciplines, such as physicians, nurses, or genetic counsellors, to ensure their patients receive the best possible care.
(6) Pharmacists should be involved in pharmacogenomics research and facilitate the development of clinical practice guidelines.
(7) Pharmacists should guide implementation efforts and educate healthcare professionals in the adoption of pharmacogenomic testing globally.

As the global consensus solidifies in favour of pharmacogenomic integration producing optimal patient care, numerous pharmaceutical organizations have begun to advocate for pharmacists to assume leadership in pharmacogenomic service implementation. Pharmacists’ roles will increasingly evolve into patient education as prescribers and patients become more familiar with pharmacogenomic testing. Therefore, enhancing pharmacist education must bridge the knowledge gap and bolster confidence in counselling patients or discussing pharmacogenomic test results with other healthcare professionals.

## Figures and Tables

**Figure 1:**
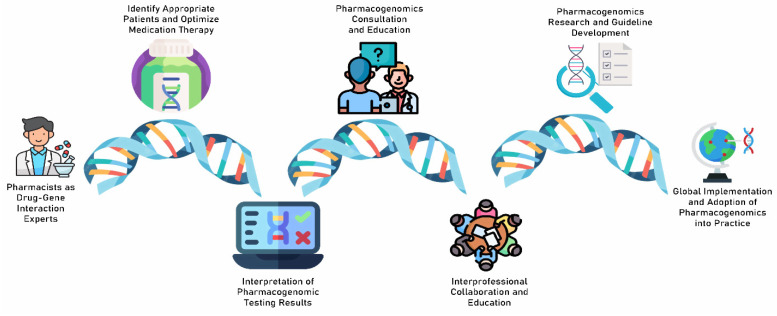
The potential roles of pharmacists in clinical pharmacogenomics.
